# Physical activity and glioma: a case–control study with follow-up for survival

**DOI:** 10.1007/s10552-022-01559-w

**Published:** 2022-02-20

**Authors:** Zohreh Basiri, Yi Yang, Fiona J. Bruinsma, Anna K. Nowak, Kerrie L. McDonald, Katharine J. Drummond, Mark A. Rosenthal, Eng-Siew Koh, Rosemary Harrup, Elizabeth Hovey, David Joseph, Geza Benke, Robyn Leonard, Robert J. MacInnis, Roger L. Milne, Graham G. Giles, Claire M. Vajdic, Brigid M. Lynch

**Affiliations:** 1 Melbourne School of Population and Global Health, The University of Melbourne, VIC, Australia; 2 Cancer Epidemiology Division, Cancer Council Victoria, 615 St Kilda Road, Melbourne, VIC 3004 Australia; 3 Medical School, QEII Medical Centre Unit, University of Western Australia, Nedlands, WA Australia; 4 Department of Medical Oncology, Sir Charles Gairdner Hospital, Perth, WA Australia; 5 Prince of Wales Clinical School, University of New South Wales, Sydney, NSW Australia; 6 Royal Melbourne Hospital, Melbourne, VIC Australia; 7 South Western Sydney Clinical School, University of New South Wales, Liverpool, NSW Australia; 8 Ingham Institute for Applied Medical Research, Sydney, NSW Australia; 9 Royal Hobart Hospital, University of Tasmania, Hobart, TAS Australia; 10 Department Medical Oncology Nelune Comprehensive Cancer Centre, Prince of Wales Hospital, University of New South Wales, Sydney, NSW Australia; 11 Department Medicine, University of New South Wales, Sydney, NSW Australia; 12 School of Medical and Health Sciences, Edith Cowan University, Joondalup, WA Australia; 13 Department of Epidemiology and Preventive Medicine, Monash University, Melbourne, VIC Australia; 14 Brain Cancer Biobanking Australia, University of Sydney, Sydney, NSW Australia; 15 Precision Medicine, School of Clinical Sciences at Monash Health, Monash University, Clayton, VIC Australia; 16 Centre for Big Data Research in Health, University of NSW, Sydney, NSW Australia; 17 Physical Activity Laboratory, Baker Heart and Diabetes Institute, Melbourne, VIC Australia

**Keywords:** Physical activity, Glioma, Case–control study, Survival

## Abstract

**Purpose:**

High-grade disease accounts for ~ 70% of all glioma, and has a high mortality rate. Few modifiable exposures are known to be related to glioma risk or mortality.

**Methods:**

We examined associations between lifetime physical activity and physical activity at different ages (15–18 years, 19–29 years, 30–39 years, last 10 years) with the risk of glioma diagnosis, using data from a hospital-based family case–control study (495 cases; 371 controls). We followed up cases over a median of 25 months to examine whether physical activity was associated with all-cause mortality. Physical activity and potential confounders were assessed by self-administered questionnaire. We examined associations between physical activity (metabolic equivalent [MET]-h/wk) and glioma risk using unconditional logistic regression and with all-cause mortality in cases using Cox regression.

**Results:**

We noted a reduced risk of glioma for the highest (≥ 47 MET-h/wk) versus lowest (< 24 METh/wk) category of physical activity for lifetime activity (OR = 0.58, 95% CI: 0.38–0.89) and at 15–18 years (OR = 0.57, 95% CI: 0.39–0.83). We did not observe any association between physical activity and all-cause mortality (HR for lifetime physical activity = 0.91, 95% CI: 0.64–1.29).

**Conclusion:**

Our findings are consistent with previous research that suggested physical activity during adolescence might be protective against glioma. Engaging in physical activity during adolescence has many health benefits; this health behavior may also offer protection against glioma.

**Supplementary Information:**

The online version contains supplementary material available at 10.1007/s10552-022-01559-w.

## Introduction

Gliomas are a heterogeneous group of primary central nervous system (CNS) tumors that originate from glial stem cells or precursor cells [1]. Rarely metastasizing beyond the CNS, glioma is generally classified as low grade (World Health Organization [WHO] grade 1 or 2) or high grade (WHO grade 3 or 4) rather than benign/malignant. Unlike most cancers, survival rates for high grade brain tumors have not improved over the last decade.

High-grade disease accounts for ~ 70% of all glioma [2–4] and although low in incidence, carries a disproportionately high mortality rate, a high social burden to both the cancer sufferer and carer, and high costs for the healthcare system. Glioblastoma is by far the most common high grade glioma and has a median survival rate of less than 15 months [5, 6]. While low grade glioma carries a much better prognosis [4, 6–11], 70% of low grade gliomas will progress to high grade glioma within 5–10 years of initial diagnosis [8].

The relatively low incidence of glioma, short survival time for the most common (grade IV) glioma grade, and high morbidity associated with the disease makes it difficult to undertake epidemiological studies to identify risk factors and any modifiable factors associated with longer survival. Gliomas are more common in older adults, men, Caucasians and individuals with some rare hereditary syndromes including neurofibromatosis (type 1 and type 2) and the tuberous sclerosis complex [12, 13]. The only well-established modifiable risk factor is ionizing radiation, but this only accounts for a small fraction of gliomas [14].

The NIH-AARP Diet and Health Study demonstrated an inverse association between physical activity at age 15–18 and risk of glioma (RR for ≥ 52 vs ≤ 12 metabolic equivalent [MET]-h/wk = 0.64; 95% CI = 0.44–0.93), however no association was found for physical activity undertaken at older ages [12]. In contrast, the European Prospective Investigation into Cancer and Nutrition (EPIC) cohort study did not find any associations between physical activity and glioma [15]. Similarly, the National Cancer Institute Cohort Consortium Physical Activity Pooling Project (which harmonized data from 1.44 million cohort study participants across the USA and Europe) did not find evidence of an association between physical activity during adulthood and brain cancer (glioma was not examined separately) [16].

Age at diagnosis, tumor grade, extent of surgical resection, performance status, and treatment undertaken are established predictors of mortality following a glioma diagnosis [17]. The influence of modifiable factors, other than treatment, on the outcome of glioma is largely unknown [18]. Only one study has examined the association between physical activity after a diagnosis of glioma and survival. Two hundred and forty-three adults with grade 3 or 4 glioma were followed for a median of 27 months; participating in ≥ 9 vs < 9 MET-h/wk after diagnosis was associated with a lower risk of death (HR = 0.64; 95% CI: 0.46–0.91) and a median survival time of 22 compared with 13 months [19]. However, observational studies of post-diagnosis physical activity in cancer survivors are prone to considerable reverse causation, and should be interpreted with caution [20].

We conducted a hospital-based family case–control study to assess the association of physical activity with both risk of glioma and mortality following a glioma diagnosis.

## Materials and methods

### Study sample

Cases were aged between 18 and 80 years, resident in one of five Australian states (New South Wales, Victoria, Queensland, Western Australia or Tasmania) and diagnosed between March, 2013 and May, 2017. Cases were recruited via collaborative clinical networks including public and private hospitals with general or specialist neuro-oncology clinics. Clinical trial sites represented the majority, or in some juridictions the only, service for the investigation and treatment of neurological cancers. Cases were diagnosed with cranial glioma low grade (grade I or II; 16%), high grade (grade III or IV; 72%) or unknown grade (11%).

**Fig. 1:**
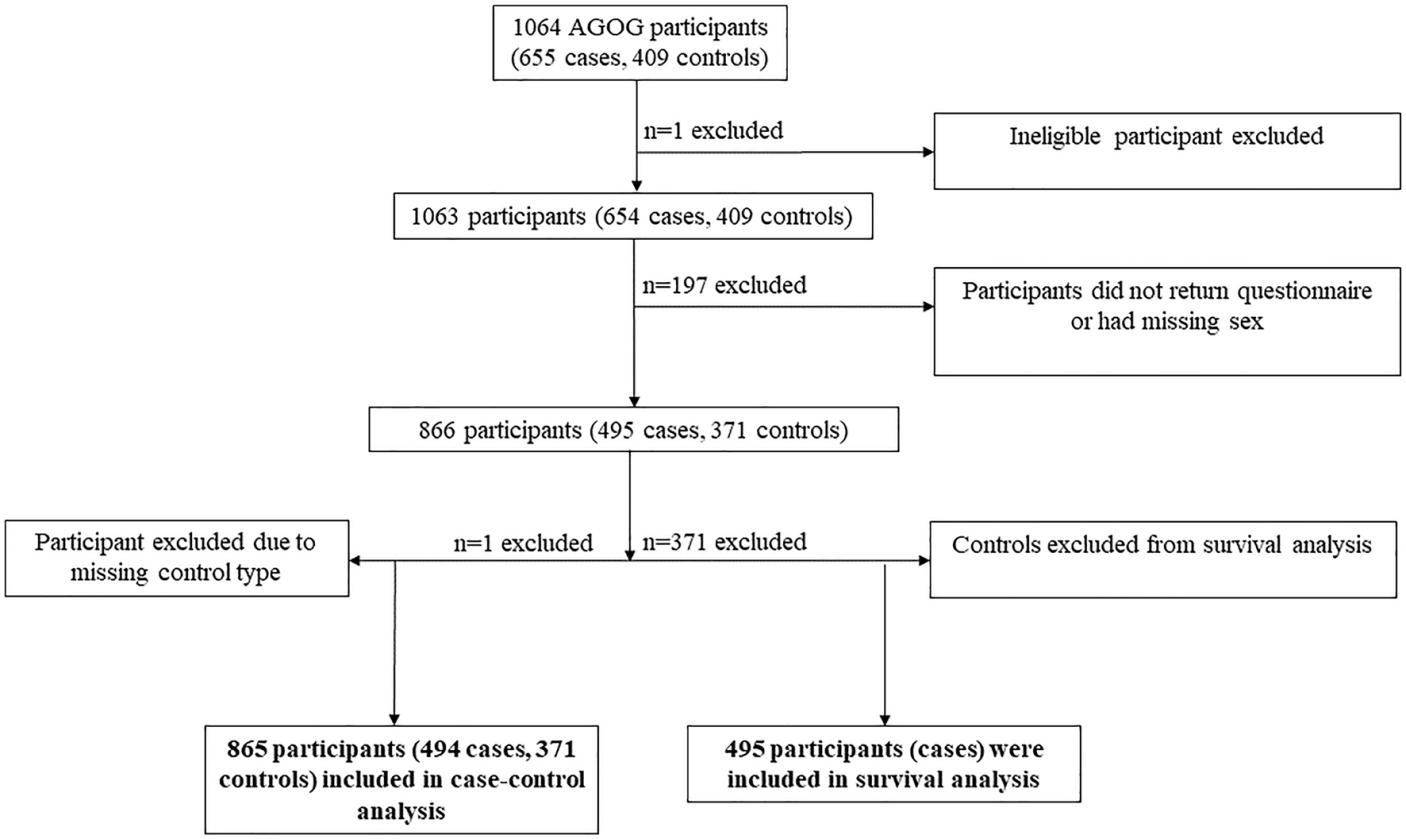
Flow of participants through the study

Controls were recruited from family members (siblings and/or partners) of cases. When a case had multiple siblings, and consented to all of them being approached, when available the sibling of the same sex and closest in age to the case was initially approached. Of those approached by the study coordinating center to participate, 172 cases and 147 controls could not be contacted. A total of 655 cases enrolled in the study (83.4%) and 130 refused (16.6%); the corresponding numbers of controls were 392 (81.0%) and 92 (19.0%). Of these, 495 cases and 371 controls fully completed the risk factor questionnaires. Figure 1 describes the recruitment to the study, for both the case–control and survival analyses.

### Data collection

Data for the study were collected by self-completed questionnaire following the construction of a ‘lifetime residence and work calendar’ to help minimize recall error.

#### Exposure variables

Participants reported the amount of time they had typically spent (h/wk) performing light-intensity physical activity (examples provided: bowling, golf (cart), table tennis, slow walking/dancing, light gardening, light housework, fishing, light calisthenics) and moderate- to vigorous-intensity physical activity (MVPA; examples provided: tennis, cycling, swimming, heavy gardening, weight lifting, running, aerobics, fast walking, heavy housework, basketball, football, rowing, fast dancing, hiking, racquetball) at ages 15–18 years, 19–29 years, 30–39 years and during the 10 years prior to diagnosis or completion of the questionnaire (controls). Seven response options were provided: never, rarely, weekly but < 1 h/wk, 1–3 h/wk, 4–7 h/wk, > 7 h/wk, and do not know.

We generated a quasi-continuous total physical activity variable in MET-h/wk for each age group using the formula: hours of light physical activity (median value within response options) * 3 (METs) + hours of moderate/vigorous activity (median value within response options) * 5 (METs). A lifetime physical activity variable was created by summing the quasi-continuous variables from applicable age periods and dividing by that number. Categorical variables were created for physical activity at each age and lifetime physical activity, based on the underlying distribution of the quasi-continuous variables in the controls: < 24 MET-h/wk (ref), 24–< 47 MET-h/wk, ≥ 47 MET-h/wk.

#### Potential confounders

We generated directed acyclic graphs (DAGs) [21] to help guide decisions for the inclusion of confounders in our multivariable models. While physical activity was reported at different ages, data on potential confounding variables were reported either for the year prior to diagnosis or at the time of interview. The variables considered as confounders for different models for glioma risk are presented in Supplementary Fig. S1. Potential confounders included: age (years); sex (male, female); ethnicity/race (Caucasian, other); income (< $50,000AUD per year, $50,000–$100,000AUD per year, > $100,000AUD per year); education attainment (completed high school, some university or vocational training, completed university degree); screen time (≤ 18 h/wk, > 18–25 h/wk, > 25 h/wk); alcohol consumption (gm/wk); and smoking status (never, former, current regular smoker at least for 12 months).

We considered body mass index (BMI) to be a mediator in our main models, but we performed sensitivity analyses to model BMI as a confounder. Because BMI had only been assessed for the year prior to diagnosis (cases) or completion of the questionnaire (controls), we only conducted this sensitivity analysis for the models examining physical activity during the last 10 years or lifetime physical activity. In our sensitivity analyses we also included potential confounders that were not assessed for the same age periods as physical activity. Education and income reflect socioeconomic position, which is often stable across the life course [22]. Similarly, drinking and smoking habits are often established in late adolescence or early adulthood [23]. For the sensitivity analyses we also adjusted for age in all models, not just those examining physical activity during the last 10 years or lifetime physical activity.

For survival analyses, we considered previous cancer diagnoses as a potential confounder. Again, BMI was primarily considered a mediator, but we adjusted for this variable in sensitivity analyses (See DAG Supplementary Fig. S2). Tables summarizing the hypothesized underlying confounding structure for the case–control and survival analyses are presented in Supplementary Tables S1 and S2.

#### All-cause mortality

Deaths were ascertained through record linkage to the Victorian Registry of Births, Deaths and Marriages, and the National Death Index at the Australian Institute of Health and Welfare. The National Death Index is a high-quality, population-based registry compiled from Registry of Births, Deaths and Marriages data supplied by each state and territory. The Australian Institute of Health and Welfare uses probabilistic record linkage based on full name, date of birth, sex, date of last contact, and address; deterministic linkage is not possible in Australia because we do not have a unique personal identification number. While vital status was ascertained for all participants, cause of death was not available for all due to some juridstictions having a delay of several years to complete adjudication.

### Statistical analysis

#### Case–control analyses

We examined the associations for the quasi-continuous physical activity measure (per 10 MET-h/wk) and the categorical measures of total physical activity at each age and over the lifetime. Primary and sensitivity analyses were adjusted for different potential confounders, as summarized in Supplementary Table 1. We used unconditional logistic regression models for an unmatched design, and applied the *vce(cluster clustvar)* option in the model to allow for clustering within families for sibling controls.

#### Survival analyses

We calculated overall survival as the number of months from diagnosis to death or last update of vital status (as one common censoring date for all patients). Cox proportional hazards regression was used to estimate multivariate HRs and 95% CI. We tested the proportional hazards assumption by including an interaction term between time and the covariates and assessed the statistical significance of the interaction terms. In the instance of violating the proportional hazards assumption, the corresponding variable remained with the interaction term in the model.

For cases and controls, there was 18% and 20% missingness in the physical activity data for the past 10 years and ages 30–39 years, and 14% and 15% for the ages 19–29 and 15–18 years. Multiple imputation by chained Eqs. (25 imputations) was used to impute missing data under the assumption that the data were missing at random [26]. All analyses were performed using STATA software version 15.1 (Stata Corp, College Station, TX).

## Results

This study involved 495 glioma cases and 371 controls; cases and controls were very similar in age and BMI, however 63% of the cases and 38% of controls were men. Table 1 presents the characteristics of the study participants.

**Table 1:** Characteristics of cases (*n* = 495) and controls (*n* = 371) in the glioma family case–control study

Participant characteristics	Cases	Controls
Participants (n)	495	371
Days between diagnosis and consent, mean (SD)	92.5 (171.3)	–
Age (years), mean (SD)	54.2 (14.5)	54.3 (13.5)
Men, *n* (%)	312 (63.0)	140 (37.7)
BMI (kg/m^2^), mean (SD)	27.7 (5.7)	27.0 (5.1)
Caucasians, *n* (%)	447 (91.0)	351 (94.9)
College education, *n* (%)	161 (32.7)	142 (38.3)
High income, *n* (%)	121 (28.7)	119 (37.7)
Current smokers, *n* (%)	47 (9.7)	23 (6.3)
Alcohol (grams/wk), median (IQR)	49 (128)	54 (139)
Physical activity in past 10 years (MET-h/wk), mean (SD)	32.2 (18.2)	34.0 (17.0)
< 24 MET-h/wk (ref) physical activity in last 10 years, *n* (%)	152 (38.4)	94 (29.9)
24–< 47 MET-h/wk physical activity in past 10 years, *n* (%)	117 (29.6)	111 (35.4)
≥ 47 MET-h/wk physical activity in past 10 years, *n* (%)	127 (32.1)	109 (34.7)
Lifetime physical activity, (MET-h/wk), mean (SD)	34.4 (15.2)	36.0 (14.7)
Screen time in one year before diagnosis (h/wk), mean (SD)	20.9 (6.4)	21.4 (6.0)

All statistics in the table are based on the complete case data prior to multiple imputation

Table 2 presents the results from the case–control study examining associations between the quasi-continuous measure of physical activity at each age and for lifetime physical activity. Lower risk of developing glioma were associated with physical activity at age 15–18 years (OR_per 10 MET-h/wk_ = 0.88, 95% CI: 0.80–0.96) and for lifetime physical activity (OR_per 10 MET-h/wk_ = 0.89, 95% CI: 0.80–0.99). Table 2 also presents the results for categories of physical activity. Lower risks of glioma were associated with the highest category of activity (≥ 47 MET-h/wk versus < 24 MET-h/wk) during adolescence (15–18 years; OR = 0.57, 95% CI: 0.39–0.83), as well as lifetime activity (OR = 0.58, 95% CI: 0.38–0.89). The results of the sensitivity analyses did not materially differ from the primary analyses (results not shown).

**Table 2:** Odds ratios and 95% confidence intervals for glioma diagnosis in relation to physical activity at different ages and lifetime physical activity in Australia (2012–2014)

	Cases in original dataset*	Cases after multiple imputation**	Model 1		Model 2	
			OR (95% CI)	*P* value	OR (95% CI)	*P* value
Previous 10 years (per 10 MET-h/wk)^a^	396	494	0.95 (0.87–1.04)	0.269	0.95 (0.87–1.04)	0.284
< 24 MET-h/wk (ref)^a^	152	191	1.00		1.00	
24–< 47 MET-h/wk	117	150	0.74 (0.52–1.06)	0.102	0.74 (0.51–1.07)	0.107
≥ 47 MET-h/wk	127	153	0.78 (0.53–1.14)	0.196	0.78 (0.53–1.14)	0.203
30–39 years of age (per 10 MET-h/wk)^b^	381	494	0.94 (0.86–1.03)	0.187	0.94 (0.86–1.03)	0.190
< 24 MET-h/wk (ref)^b^	123	163	1.00		1.00	
24–< 47 MET-h/wk	143	183	1.05 (0.71–1.54)	0.818	1.04 (0.71–1.55)	0.826
≥ 47 MET-h/wk	115	148	0.71 (0.48–1.05)	0.086	0.71 (0.48–1.04)	0.082
19–29 years of age (per 10 MET-h/wk)^b^	415	494	0.92 (0.84–1.01)	0.082	0.92 (0.84–1.01)	0.069
< 24 MET-h/wk (ref)^b^	108	132	1.00		1.00	
24–< 47 MET-h/wk	166	197	1.05 (0.71–1.56)	0.808	1.04 (0.70–1.55)	0.846
≥ 47 MET-h/wk	141	165	0.73 (0.49–1.08)	0.116	0.72 (0.48–1.07)	0.102
15–18 years of age (per 10 MET-h/wk)^c^	408	494	0.88 (0.80–0.96)	0.003	0.88 (0.81–0.96)	0.006
< 24 MET-h/wk (ref)^c^	133	164	1.00		1.00	
24–< 47 METh/wk	125	155	0.74 (0.51–1.06)	0.101	0.79 (0.54–1.16)	0.227
≥ 47 MET-h/wk	150	175	0.57 (0.39–0.83)	0.003	0.59 (0.40–0.87)	0.007
Lifetime activity (per 10 MET-h/wk)^a^	334	494	0.89 (0.80–0.99)	0.031	0.89 (0.80–0.99)	0.033
< 24 MET-h/wk (ref)^a^	93	140	1.00		1.00	
24–< 47 MET-h/wk	151	237	0.82 (0.55–1.21)	0.321	0.82 (0.55–1.22)	0.322
≥ 47 MET-h/wk	90	117	0.58 (0.38–0.89)	0.012	0.58 (0.38–0.89)	0.013

^*^Number of cases in the unimputed dataset

^**^Averaged number of cases in each imputed dataset included in the analysis

^a^Model 1 adjusted for age, sex, ethnicity, income, education, screen time, smoking, alcohol; model 2 additionally adjusted for BMI

^b^Model 1 adjusted for sex, ethnicity, income, education; model 2 additionally adjusted for age, screen time, smoking and alcohol

^c^Model 1 adjusted for sex, ethnicity; model 2 additionally adjusted for age, income, education, screen time, smoking and alcohol

^d^Model 1 adjusted for sex, ethnicity; model 2 additionally adjusted for age, alcohol, smoking, income, education, screen time

Cases were followed up for a median of 25 months, with an interquartile range of 14–43 months. Cause of death was available for 412 cases; 401 deaths were due to brain cancer (97%). Table 3 presents results from Cox proportional hazards regression models, which show that pre-diagnosis physical activity was not associated with all-cause mortality at any time period (Table 3). The results of the sensitivity analyes did not materially differ from the primary analyses (results not shown).

**Table 3:** Hazard ratios and 95% confidence intervals for all-cause mortality following glioma diagnosis in relation to physical activity at different ages and lifetime physical activity

	No. deaths/person months	Model 1		Model 2	
		HR (95% CI)	*P* value	HR (95% CI)	*P* value
Past 10 years (10 MET-h/wk)^a^	332/14,998	0.97 (0.91–1.04)	0.335	0.99 (0.92–1.06)	0.725
< 24 MET-h/wk (ref)^a^	131/5,786	1.00		1.00	
24—< 47 MET-h/wk	102/4,369	0.91 (0.68–1.23)	0.542	0.96 (0.71–1.30)	0.776
≥ 47 MET-h/wk	99/4,844	0.89 (0.67–1.18)	0.420	0.95 (0.71–1.27)	0.739
30–39 years of age^b^	332/1,4998	1.00 (0.93–1.07)	0.958	0.96 (0.90–1.03)	0.284
< 24 MET-h/wk (ref)^b^	116/4,834	1.00		1.00	
24–< 47 MET-h/wk	119/5,693	0.85 (0.64–1.13)	0.269	0.75 (0.56–1.01)	0.060
≥ 47 MET-h/wk	97/4,472	0.96 (0.71–1.29)	0.776	0.83 (0.61–1.13)	0.244
19–29 years of age^c^	332/14,998	0.99 (0.93–1.06)	0.747	0.99 (0.92–1.06)	0.727
< 24 MET-h/wk (ref)^c^	88/4,129	1.00		1.00	
24–< 47 MET-h/wk	140/5,738	1.18 (0.89–1.56)	0.264	1.20 (0.90–1.60)	0.211
≥ 47 MET-h/wk	104/5,131	1.02 (0.75–1.39)	0.896	1.03 (0.75–1.42)	0.851
15–18 years of age^d^	332/14,998	0.98 (0.92–1.05)	0.599	1.01 (0.95–1.08)	0.715
< 24 MET-h/wk (ref)^d^	117/5,059	1.00		1.00	
24–< 47 MET-h/wk	99/4,744	0.85 (0.64–1.13)	0.256	0.92 (0.68–1.24)	0.583
≥ 47 MET-h/wk	116/5,195	0.94 (0.71–1.23)	0.634	1.09 (0.81–1.47)	1.578
Lifetime activity^a^	332/14,998	0.98 (0.90–1.06)	0.590	0.99 (0.92–1.08)	0.865
< 24 MET-h/wk (ref)^a^	98/4,398	1.00		1.00	
24–< 47 MET-h/wk	162/6,804	1.02 (0.71–1.37)	0.870	1.07 (0.80–1.43)	0.659
≥ 47 MET-h/wk	72/3,796	0.91 (0.64–1.29)	0.606	0.98 (0.68–1.39)	0.893

^*^Number of deaths/person months is the average across 25 multiple imputed datasets

^a^Model 1 adjusted for age, sex, ethnicity, income, education, alcohol, smoking, personal cancer history (excluding non-melanoma skin cancer) and screen time; model 2 additionally adjusted for BMI

^b^Model 1 adjusted for sex, ethnicity, income, education and personal cancer history (excluding non-melanoma skin cancer); model 2 additionally adjusted for age, alcohol, smoking and screen

^c^Model 1 adjusted for sex, ethnicity, income, education; model 2 additionally adjusted for age, alcohol, smoking and screen time

## Discussion

We used a hospital-based family case–control study to examine associations between physical activity performed at different ages and intensity and lifetime physical activity and glioma risk. Greater physical activity at age 15–18 years, and over the lifetime was associated with lower risk of developing glioma. Pre-diagnosis physical activity was not associated with all-cause mortality for cases.

Recruitment to the case–control study was challenging, due to the morbidity and mortality associated with glioma and its treatment. Given that the median survival for (the most common high grade glioma) is less than 15 months [5, 6], and that it took three months on average to consent cases who participated in this study, it is highly likely that our sample does not include people diagnosed with the most aggressive or difficult to manage gliomas. This selection bias will have affected both the case–control and survival analyses. We accounted for differences between genetically related controls (siblings) and non-genetically related controls (partners and other controls) in our analyses, and the use of different types of controls did not affect the results. Other methodological challenges may also have introduced bias. Adults generally overestimate their physical activity compared with estimates derived from accelerometry [27]. We asked participants to recall the type, frequency and duration of physical activity they performed decades ago. Although we used a ‘lifetime residence and work calendar’ to help prompt memory, substantial recall error was likely. As a result, the study findings may have been affected by non-differential and differential measurement error.

A number of the confounding factors were not available for each time period. While some would remain relatively stable across the life course (e.g., educational attainment), some may change considerably over time. It would have been useful to have measures of BMI for the different time periods at which physical activity was reported, given the hypothesis that early life energy balance (affected by both BMI and physical activity) is a risk factor for glioma. The BMI estimate at time of interview (for cases) might have been affected by dexamethasone (steroid) use for the management of their disease.

We observed a strong and significant association between physical activity during adolescence and lower glioma risk. This is consistent with findings from the NIH-AARP Diet and Health Study, which demonstrated an inverse association between physical activity at age 15–18 and risk of glioma [12]. The point estimate presented by Moore et al. for the highest vs. lowest category of physical activity between ages 15 to 18 years was 0.64 (95% CI: 0.43–0.94), which is very similar to our result (OR = 0.57, 95% CI: 0.39–0.83). Both the NIH-AARP Diet and Heath Study and ours used a broad and inclusive measure of physical activity, assessing light and moderate-vigorous activities not restricted to any behavioral domain. The consistency of these findings supports the premise that being highly active—regardless of type or intensity of activity—during adolescence may reduce glioma risk.

Other research on physical activity and brain/central nervous system tumors is limited, and contradictory. In the Million Women cohort study, an inverse association between strenuous exercise and incidence of all central nervous system tumors, meningioma and glioma was observed [28]. In contrast, neither the EPIC cohort study [15] nor the National Cancer Institute Cohort Consortium Physical Activity Pooling Project [16] reported evidence of an association between physical activity during adulthood and brain cancer.

It has been proposed that the associations of body mass, height and physical activity with risk of glioma may be related to early life energy balance, and subsequent influences on circulating insulin levels [12]. Hyper-insulinemia may be caused by obesity and low levels of physical activity. Insulin is known to have a promitotic effect and in vivo experiments have shown that dietary hyperinsulinemia is associated with cell proliferation and tumor growth [29]. Insulin increases the levels of free circulating insulin-like growth factor-1 (IGF-1) in the body by binding to the receptors of IGF-1 [12, 29]. IGF-1 plays a crucial role as a neurotrophic factor in the early development of the peripheral and central nervous systems [12, 30]. Considering the role of IGF-1 in the proliferation, differentiation and apoptosis of glial cells, it is biologically plausible that physical inactivity, including in earlier life, may contribute to glioma risk.

To date only one study has examined physical activity and mortality after a glioma diagnosis. Ruden et al. reported a reduced risk associated with post-diagnosis physical activity [19], but the robustness of these findings is challenged by reverse causation. Our findings suggest that physical activity performed across the life course is not associated with mortality following a glioma diagnosis. Previous research has shown that pre-morbid obesity was associated with reduced overall survival in patients with high-grade glioma [18], supporting the concept that energy balance may play a role in survival. Further, it is increasingly understood that many chronic diseases, which contribute to mortality rates in populations affected by cancer, begin developing in childhood and adolescence, highlighting the need to consider a life course approach [31]. Therefore further research (ideally, randomized-controlled trials) is crucial to clarify whether physical activity can help extend survival for glioma patients.

## Conclusion

Few studies have explored associations between physical activity and glioma, a relatively uncommon cancer. Our study suggests that physical activity, particularly during adolescence, may reduce the risk of glioma later in life. Pre-diagnosis physical activity did not appear to influence survival after a glioma diagnosis. These findings strengthen the argument that physical inactivity is an important and modifiable glioma risk factor to be addressed by public health interventions.

## Supplementary Information

Below is the link to the electronic supplementary material.

Supplementary file1 (PDF 336 KB)

## Data Availability

Data can be supplied upon reasonable request.

## Code availability

Code can be supplied upon reasonable request.

## References

[1] WHO Classification of Tumours of the Central Nervous System, WHO Classification of Tumours, In: Louis DN, Ohgaki H, Wiestler OD, Cavenee WK (eds), 4th Edn, Vol. 1. Lyon, France: IARC Press

[2] Bauchet L, Rigau V, Mathieu-Daudé H (2007). French brain tumor data bank: methodology and first results on 10,000 cases. J Neurooncol.

[3] Chang SM, Parney IF, Huang W (2005). Patterns of care for adults with newly diagnosed malignant glioma. JAMA.

[4] Dolecek TA, Propp JM, Stroup NE, Kruchko C (2012) CBTRUS Statistical Report: Primary brain and central nervous system tumors diagnosed in the United States in 2005–2009. Neuro Oncol 14(Suppl 5):v1–v49. 10.1093/neuonc/nos218 10.1093/neuonc/nos218 PMC3480240 23095881

[5] Laws ER, Parney IF, Huang W (2003). Survival following surgery and prognostic factors for recently diagnosed malignant glioma: data from the glioma outcomes project. J Neurosurg.

[6] Rosenthal MA, Drummond KJ, Dally M (2006). Management of glioma in Victoria (1998–2000): retrospective cohort study. Med J Aust.

[7] Cairncross JG (2000). Understanding low-grade glioma: a decade of progress. Neurology.

[8] Maher EA, Furnari FB, Bachoo RM (2001). Malignant glioma: genetics and biology of a grave matter. Genes Dev.

[9] McDonald KL, O'Sullivan MG, Parkinson JF (2007). IQGAP1 and IGFBP2: valuable biomarkers for determining prognosis in glioma patients. J Neuropathol Exp Neurol.

[10] Olson JD, Riedel E, DeAngelis LM (2000). Long-term outcome of low-grade oligodendroglioma and mixed glioma. Neurology.

[11] Surawicz TS, McCarthy BJ, Kupelian V, Jukich PJ, Bruner JM, Davis FG (1999). Descriptive epidemiology of primary brain and CNS tumors: results from the Central Brain Tumor Registry of the United States, 1990–1994. Neuro Oncol.

[12] Moore SC, Rajaraman P, Dubrow R (2009). Height, body mass index, and physical activity in relation to glioma risk. Cancer Res.

[13] Reuss D, von Deimling A (2009). Hereditary tumor syndromes and gliomas. Recent Results Cancer Res.

[14] Molinaro AM, Taylor JW, Wiencke JK, Wrensch MR (2019). Genetic and molecular epidemiology of adult diffuse glioma. Nat Rev Neurol.

[15] Michaud DS, Bové G, Gallo V (2011). Anthropometric measures, physical activity, and risk of glioma and meningioma in a large prospective cohort study. Cancer Prev Res.

[16] Moore SC, Lee IM, Weiderpass E (2016). Association of leisure-time physical activity with risk of 26 types of cancer in 1.44 million adults. JAMA Intern Med.

[17] Stupp R, Hegi ME, Gilbert MR, Chakravarti A (2007). Chemoradiotherapy in malignant glioma: standard of care and future directions. J Clin Oncol.

[18] Siegel EM, Nabors LB, Thompson RC (2013). Prediagnostic body weight and survival in high grade glioma. J Neurooncol.

[19] Ruden E, Reardon DA, Coan AD (2011). Exercise behavior, functional capacity, and survival in adults with malignant recurrent glioma. J Clin Oncol.

[20] Lynch BM, Leitzmann MF (2017). An evaluation of the evidence relating to physical inactivity, sedentary behavior, and cancer incidence and mortality. Curr Epidemiol Rep.

[21] VanderWeele TJ, Robins JM (2007). Directed acyclic graphs, sufficient causes, and the properties of conditioning on a common effect. Am J Epidemiol.

[22] Hossin MZ, Björk J, Koupil I (2020). Early-life social and health determinants of adult socioeconomic position: associations and trends across generations. J Epidemiol Community Health.

[23] Australian Institute of Health and Welfare (2020). Australia’s children.

[24] Mansournia MA, Jewell NP, Greenland S (2018). Case-control matching: effects, misconceptions, and recommendations. Eur J Epidemiol.

[25] Pearce N (2016). Analysis of matched case-control studies. BMJ..

[26] White IR, Royston P, Wood AM (2011). Multiple imputation using chained equations: issues and guidance for practice. Stat Med.

[27] Sylvia LG, Bernstein EE, Hubbard JL, Keating L, Anderson EJ (2014). Practical guide to measuring physical activity. J Acad Nutr Diet.

[28] Benson VS, Pirie K, Green J, Casabonne D, Beral V (2008). Lifestyle factors and primary glioma and meningioma tumours in the Million Women Study cohort. Br J Cancer.

[29] Peeters PJ, Bazelier MT, Leufkens HG, de Vries F, De Bruin ML (2015). The risk of colorectal cancer in patients with type 2 diabetes: associations with treatment stage and obesity. Diabetes Care.

[30] Bianchi VE, Locatelli V, Rizzi LJ (2017). Neurotrophic and neuroregenerative effects of GH/IGF1. IJMS.

[31] Committee on Physical Activity and Physical Education in the School Environment. , Kohl HI, Cook H (2013). Educating the student body: taking physical activity and physical education to school. Washington.

