# Supplementary material for: Physical activity and glioma: a case–control study with follow-up for survival
Source: Cancer Causes Control. 2022 Feb 20;33(5):749–57. doi: 10.1007/s10552-022-01559-w (PMC9010385; doi:10.1007/s10552-022-01559-w)
Supplement: Supplementary file 1 — Supplementary file1 (PDF 336 KB) [file 10552_2022_1559_MOESM1_ESM.pdf]

Basiri *et al*. Supplementary Material

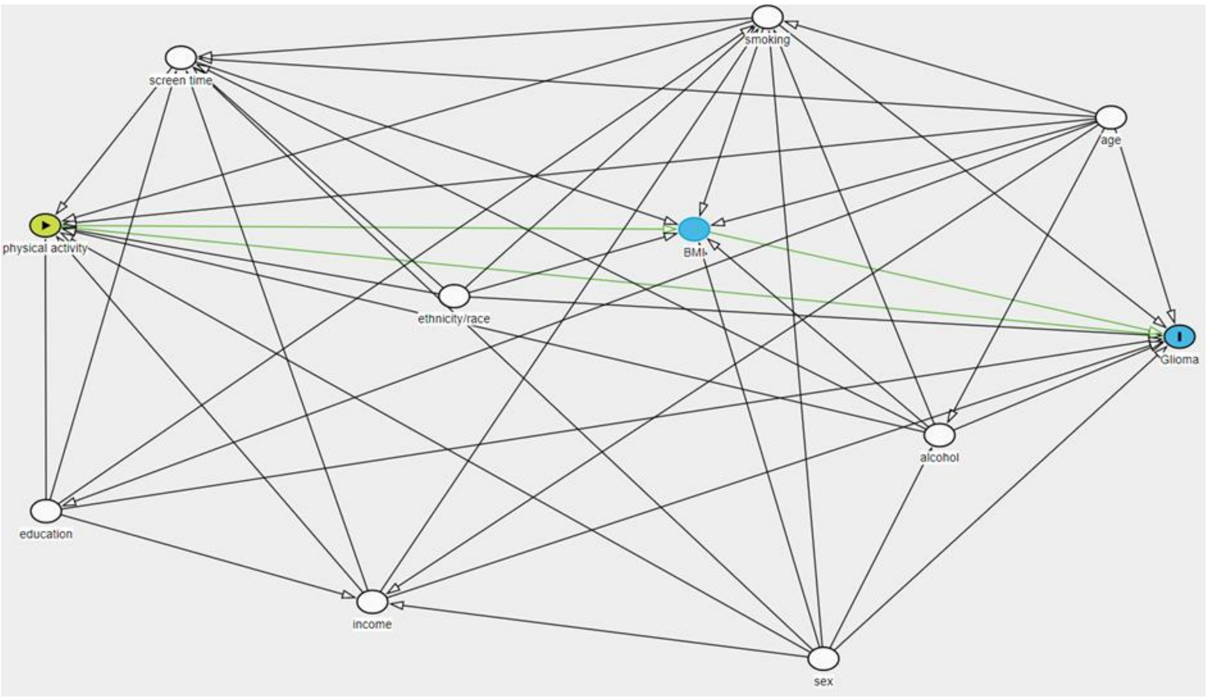

Supplementary Figure 1. Directed acyclic graph, presenting potential confounders in the association of physical activity in the last 10 years and risk of glioma.

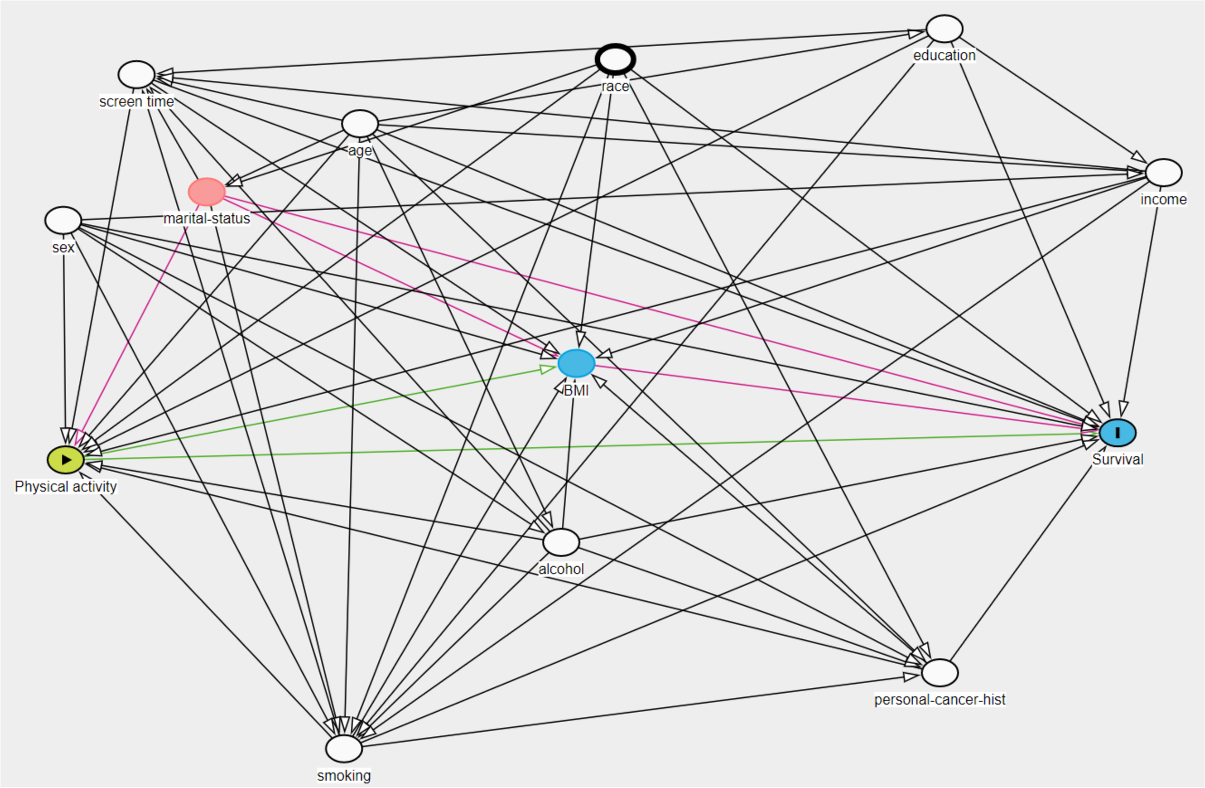

Supplementary Figure 2. Directed acyclic graph, presenting potential confounders in the association of physical activity in the past 10 years and survival of glioma patients.

Supplementary Table 1. Adjusted variables in the main models and sensitivity analyses in the case-control analysis

Exposures	Covariates in case-control analysis main models	Added covariates in case-control sensitivity analysis
**Physical activity in the past 10 years**	Age, sex, ethnicity, income, education, alcohol, smoking, screen time	BMI
**Physical activity at ages 30-39**	Sex, ethnicity, income, education	Age, alcohol, smoking, screen time
**Physical activity at ages 19-29**	Sex, ethnicity, income, education	Age, alcohol, smoking, screen time
**Physical activity at ages 15-18**	Sex, ethnicity	Age, alcohol, smoking, income, education, screen time
**Lifetime physical activity**	Age, sex, ethnicity, income, education, alcohol, smoking, screen time	BMI

Supplementary Table 2. Adjusted variables in the main models and sensitivity analyses in the survival analysis

Exposures	Covariates in survival analysis main models	Added covariates in survival analysis sensitivity analysis
**Physical activity in the past 10 years**	Age, sex, ethnicity, income, education, alcohol, smoking, cancer history, screen time	1. BMI<br>2. Grade<br>3. Excluded those with gap >=12months*
**Physical activity at ages 30-39**	Sex, ethnicity, income, education, cancer history	Age, alcohol, smoking, screen time
**Physical activity at ages 19-29**	Sex, ethnicity, income, education	Age, alcohol, smoking, screen time
**Physical activity at ages 15-18**	Sex, ethnicity	Age, alcohol, smoking, income, education, screen time
**Lifetime physical activity**	Age, sex, ethnicity, income, education, alcohol, smoking, cancer history, screen time	BMI

*****Participants who there was a gap greater or equal to one year between their diagnosis and participation date were excluded from analysis.
